# Advanced imaging–integrated rTMS therapy for frontal network recovery after traumatic brain injury

**DOI:** 10.3389/fnhum.2026.1900612

**Published:** 2026-08-25

**Authors:** Nan Hu, Zhiling Chen, Jiante Li, Yang Zheng

**Affiliations:** 1 Department of Anesthesiology, Tianjin Medical University Cancer Institute and Hospital, National Clinical Research Center for Cancer, Key Laboratory of Cancer Prevention and Therapy, Tianjin, China; 2 Fuzong Clinical Medical College of Fujian Medical University, Fuzhou, China; 3 Department of Neurosurgery, The 900th Hospital of the People's Liberation Army Joint Logistics Support Force, Fuzhou, China; 4 Department of Anorectal Surgery, The Second Affiliated Hospital and Yuying Children’s Hospital of Wenzhou, Medical University, Wenzhou, China; 5 Emergency and Critical Care Center, Intensive Care Unit, Zhejiang Provincial People’s Hospital, Affiliated People’s Hospital, Hangzhou Medical College, Hangzhou, Zhejiang, China

**Keywords:** dorsolateral prefrontal cortex, executive function, multimodal neuroimaging, repetitive transcranial magnetic stimulation, traumatic brain injury

## Abstract

**Background:**

Traumatic brain injury is frequently followed by persistent executive dysfunction linked to disruption of frontal and frontoparietal networks. High-frequency repetitive transcranial magnetic stimulation (rTMS) targeting the dorsolateral prefrontal cortex (DLPFC) has been proposed as a potential adjunctive intervention to support frontal-network recovery. Still, evidence remains heterogeneous and biomarker-guided interpretation is limited.

**Methods:**

This retrospective multicenter observational cohort study evaluated adults with traumatic brain injury and persistent executive dysfunction who received standard neurorehabilitation with or without adjunctive high-frequency rTMS. Patients were classified into an rTMS-exposed cohort and a non-rTMS comparison cohort according to documented treatment exposure. Primary outcomes were changes in Frontal Assessment Battery (FAB) and MoCA executive-domain scores. Secondary outcomes included Glasgow Outcome Scale–Extended and Trail-Making Test A/B performance. Multimodal biomarkers included TMS-EEG N100/P200 amplitudes, fNIRS task-evoked left DLPFC oxyhemoglobin (HbO2) response, and resting-state fMRI DLPFC–frontoparietal connectivity. Adjusted regression analyses were performed to estimate associations between documented rTMS exposure and study outcomes.

**Results:**

Forty-eight patients were included, with 24 in the rTMS-exposed cohort and 24 in the non-rTMS comparison cohort. Documented rTMS exposure was associated with greater improvement in FAB score at post-treatment assessment (+3.1 points; 95% CI, 1.6 to 4.6; *p* < 0.001) and MoCA executive-domain score (+2.8 points; 95% CI, 1.3 to 4.3; *p* = 0.001). Functional recovery and Trail-Making Test performance also favored the rTMS-exposed cohort. Biomarker analyses showed greater N100/P200 modulation, increased left DLPFC HbO response, and stronger DLPFC–frontoparietal connectivity. Observed biomarker changes were correlated with executive-function improvement; however, these associations should be interpreted as exploratory and hypothesis-generating given the observational design and limited sample size. Adverse events were mild, with no documented seizures or serious adverse events.

**Conclusion:**

High-frequency left-DLPFC rTMS exposure was associated with improved executive recovery and measurable frontal-network modulation after traumatic brain injury. These findings suggest that multimodal neurophysiological and imaging biomarkers may provide useful measures of target engagement for future prospective studies. Given the retrospective observational design, modest sample size, and short-term follow-up, causal inferences regarding treatment effects or biomarker-guided neurorehabilitation cannot be established.

## 1 Introduction

Traumatic brain injury (TBI) is one of the most prevalent long-term neurological conditions globally (Obasa et al., 2024; Zhan et al., 2024). It is a condition that is increasing in incidence, prevalence and loss of healthy life years. Persistent executive dysfunction, ranging from attention and working memory to cognitive flexibility and planning, has been associated with the disruption of frontal lobe systems and frontoparietal networks, and accordingly, the dorsolateral prefrontal cortex (DLPFC) is a biologically plausible therapeutic target (Obasa et al., 2024). Repetitive transcranial magnetic stimulation (rTMS) can modulate the excitability of the cortex and large-scale connectivity in a frequency-, intensity-, and site-dependent manner, and contemporary consensus statements describe the limits of safety and dosing parameters for clinical applications (Trandafir et al., 2026).

Clinical evidence supporting rTMS after TBI remains mixed and inconclusive (Lindsey et al., 2023). One randomized, sham-controlled, crossing-over trial in mild–moderate TBI found that high-frequency rTMS of the prefrontal cortex was associated with reduction in post-concussive symptoms and EEG changes, but with only minor, objective cognitive improvement (Franke et al., 2022). However, a randomized study on chronic diffuse axonal injury did not show significant cognitive effects of left-DLPFC rTMS, reflecting the variability in rTMS timing, protocols, and outcome measures (Calderone et al., 2024). Recent syntheses propose that non-invasive brain stimulation, especially when combined with structured cognitive rehabilitation, may be beneficial in selected patients, while emphasizing the need for biomarker-informed prospective studies to understand better treatment responsiveness and underlying neurophysiological change (Nousia et al., 2022).

Multimodal neurophysiology provides readouts of objective target engagement and network repair. TMS-EEG offers direct measures of cortical reactivity and inhibition—the N100 component, which is related to GABA_B mechanisms, is robust, and research is emerging on the use of this measure as a treatment-response marker (Prokop-Millar et al., 2025). Functional near-infrared spectroscopy (fNIRS) for verbal fluency measures changes in HbO2 in the prefrontal cortex in response to the task (Kim et al., 2022). It has been shown to be modulated by rTMS in clinical populations (Zhang et al., 2024). Frontoparietal connectivity is a candidate measure of large-scale network restoration after transient stimulation, measured by resting-state fMRI (Bouchard et al., 2023). A second set of converging evidence from disorders of consciousness further suggests that prefrontal rTMS can enhance behaviorally responsiveness and reorganize connectivity. However, the extent to which these observations generalize to TBI remains uncertain (Vitello et al., 2023).

Previous rTMS studies in TBI have been inconsistent and failed to report cognitive effects, which may be due to the lack of complementary physiological assessment of target engagement and network-level effects following rTMS. We thus decided to use a multimodal neurophysiology approach, including TMS-EEG, task-evoked fNIRS and resting-state fMRI. We used these three readouts to (a) ensure on-target physiological modulation of DLPFC, (b) capture fast electrophysiological change (TMS-EEG) and slower hemodynamic reorganization (fNIRS and fMRI), and (c) relate physiological change to clinically meaningful executive gains. Accordingly, we conducted a retrospective multicenter observational cohort study to examine whether documented left-DLPFC rTMS exposure was associated with executive-function recovery and frontal-network neurophysiological measures among adults with TBI 3–12 months after injury. By integrating TMS-EEG, fNIRS, and resting-state fMRI, this study sought to explore potential associations between multimodal biomarkers and clinical outcomes that may inform future prospective biomarker-based investigations.

## 2 Materials and methods

### 2.1 Study design

This was a retrospective multicenter observational cohort study to examine the association of clinically administered high-frequency (rTMS) in patients with traumatic brain injury and frontal-network recovery. Patients in the study were patients with chronic executive dysfunction in the subacute to chronic recovery phase treated as part of standard clinical practice with standard neurorehabilitation either with or without rTMS therapy.

The main aim was to assess whether documented exposure to high-frequency rTMS over the left DLPFC was associated with changes in executive function compared with standard neurorehabilitation alone. The secondary objective was to determine whether multimodal neurophysiological and neuroimaging measures were associated with documented rTMS exposure and clinical outcomes. No causal inference regarding physiological mechanisms was intended because of the retrospective observational study design. Because treatment allocation was determined by routine clinical practice rather than randomization, the study was designed to evaluate associations between documented rTMS exposure and study outcomes rather than treatment efficacy. Residual confounding and selection bias cannot be excluded.

### 2.2 Study setting and data sources

Data were retrospectively collected from four tertiary referral centers with expertise in traumatic brain injury management, neurorehabilitation, non-invasive brain stimulation, and advanced neurophysiological assessment. Eligible patient records were identified through electronic medical records, neurorehabilitation charts, rTMS treatment logs, neuropsychological assessments, and archived multimodal neurophysiological and neuroimaging databases. Demographic information, injury characteristics, rehabilitation exposure, rTMS treatment parameters, clinical outcomes, and available biomarker data were systematically extracted using a standardized data collection framework. Clinical records were also reviewed for injury-related variables, including Glasgow Coma Scale (GCS) score, mechanism of injury, time since injury, neuroimaging findings (where available), and history of neurosurgical intervention. These variables were extracted to characterize the study population and were incorporated into adjusted analyses when sufficiently complete and reliably documented.

Demographic data, injury information, rehabilitation exposure, rTMS treatment parameters, cognitive and functional outcomes, and biomarker measures available were included in the data extracted from the dataset. If available, archived TMS-EEG and fNIRS (fNIRS) records were reviewed, as well as resting-state functional MRI (rs-fMRI). All clinical and biomarker data were arranged in a series of pre-specified retrospective time frames of baseline, post-treatment evaluation, and short-term follow-up.

If a patient was exposed to rTMS, the baseline was the closest clinical assessment prior to the first rTMS session. In the non-rTMS comparison group, baseline was considered the first qualifying neurorehabilitation assessment in the same post-injury recovery window.

### 2.3 Participants and eligibility criteria

The inclusion criteria for patients were: adults, age 18–70 years, with a documented history of closed-head traumatic brain injury (TBI) and executive dysfunction (EF) during the subacute to chronic recovery period. To be eligible, patients had to have both baseline and post-treatment cognitive evaluations, extensive documentation of rehabilitation exposure, and clinical records sufficient for the development of rTMS-exposed and non-rTMS comparison cohorts.

Patients were considered eligible if they were 3–12 months into the injury at baseline, and exhibited a history of executive dysfunction on standardized cognitive testing or were previously documented in a neuropsychological report. Existing FAB scores determined executive functioning, MoCA executive domain scores or documented clinical findings in the clinical record. Available scores of the FAB or the MoCA executive domain scores or equivalent neuropsychological documentation in the clinical file determined executive dysfunction. These measures were chosen because they directly measure frontal executive function, the primary clinical domain of interest in this study.

Where available, baseline clinical characteristics, including Glasgow Coma Scale (GCS) score at presentation, mechanism of injury, duration of post-traumatic amnesia, injury severity classification, lesion location, diffuse axonal injury (DAI), structural neuroimaging findings, and history of neurosurgical intervention, were extracted from medical records. These variables were used to characterize the study population and were considered for adjusted analyses when sufficiently documented across participating centers.

Patients were excluded if there were missing primary outcome data, uncertainty regarding treatment exposure, inadequate documentation of the intensity of the rehabilitation, or severe aphasia or profound cognitive impairment made the clinical data assessment unreliable. Other exclusion criteria were a recent history of seizures unrelated to TBI, presence of skull defects over the stimulation site, instability in neurologic/psychiatric condition, significant change in medication in the observation period, or MRI contraindications for imaging outcomes. Participants with incomplete or poor-quality TMS-EEG, fNIRS, or resting-state fMRI datasets were excluded only from the corresponding biomarker analyses while remaining eligible for primary clinical outcome analyses.

### 2.4 Exposure definition: rTMS therapy

Main exposure was high-frequency rTMS over the left DLPFC in routine neurorehabilitation treatment. The clinical records were used to determine patients’ exposure to rTMS, and patients who had a documented course of rTMS delivered to the left prefrontal cortex in addition to standard multidisciplinary rehabilitation were considered rTMS-exposed.

The following treatment parameters were taken from the rTMS treatment logs: stimulation site, stimulation frequency, stimulation intensity, pulses per session, completed sessions, and treatment duration. High frequency (10 Hz) left DLPFC stimulation was followed by the standard stimulation protocol: 2,500 pulses per session, stimulation intensity based on resting motor threshold, repeated over 4 weeks of treatment. These parameters were chosen to represent the standardized high-frequency left-DLPFC stimulation protocol recorded in the participating centers. Localization of the left DLPFC was performed using the Beam-F3 scalp-based localization method or MRI-guided neuronavigation, depending on the routine clinical practice at each participating center. The Beam-F3 method was used in 14 participants, whereas MRI-guided neuronavigation was used in 10 participants. The targeting approach used for each participant was documented where available and considered during data interpretation because differences in targeting methodology may influence stimulation accuracy and treatment response.

### 2.5 Comparison group

The comparison group was patients with traumatic brain injury who were eligible for rTMS and received standard multidisciplinary neurorehabilitation during the same clinical period. To increase comparability in clinical settings, rehabilitation practice, assessment procedures, and documentation of follow-up, these patients were identified from the same participating centers. The comparison cohort was not considered a sham-control group. Instead, it was a cohort of non-rTMS real-world rehabilitation. Clinically indicated per-patient physical, occupational, cognitive, speech-language and other supports for neurorehabilitation were provided as standard rehabilitation. To be eligible for the comparison cohort, patients met the same inclusion criteria as the rTMS-exposed cohort, with the exception of rTMS therapy. The frequency and duration of rehabilitation were taken from clinical records. To minimize treatment-selection bias, baseline demographic, injury-related, and rehabilitation variables were compared between cohorts using standardized mean differences (SMDs), and propensity score-adjusted sensitivity analyses were performed.

### 2.6 Clinical outcomes

Clinical outcomes were obtained from the neuropsychology assessment records and follow-up rehabilitation records. The most important clinical outcome was the change in executive function from baseline to post-treatment examination as assessed by the FAB and the executive-domain score of the Montreal Cognitive Assessment (MoCA). The outcomes were chosen because they were related to the study’s focus on persistent frontal executive dysfunction following a traumatic brain injury. Additional secondary outcomes were functional recovery and processing speed. The Glasgow Outcome Scale measured functional recovery, extended processing speed, and cognitive flexibility using the Trail-Making Test A and Trail-Making Test B. If present, self-reported cognitive problems were also identified as supporting outcomes. The outcomes were chosen to represent the main cognitive and functional areas of post-traumatic executive dysfunction: frontal executive control, global functional recovery, processing speed, and cognitive flexibility. Outcome measures were divided into *a priori* time periods: baseline, post-treatment, and short-term follow-up. Post-treatment assessment was defined as the assessment performed within 7 days after completion of the four-week treatment window, and short-term follow-up was defined as the assessment performed 4 weeks (±7 days) after treatment completion. The closest assessment prior to rTMS commencement was used as a baseline for rTMS-exposed patients. In comparison to patients, the baseline was set as the first assessment in the same recovery period. Because executive recovery following TBI may continue over several months, all outcome analyses were adjusted for baseline executive-function severity and time since injury whenever data were available.

### 2.7 Multimodal neurophysiological and imaging measures

Archived multimodal neurophysiological and neuroimaging datasets were analyzed when available and when quality-control criteria were satisfied. These modalities included TMS-EEG, fNIRS (fNIRS), and resting-state functional MRI (rs-fMRI) to evaluate cortical reactivity, task-related prefrontal activation, and functional network connectivity.

#### 2.7.1 TMS-EEG acquisition and preprocessing

EEG was recorded from a 64-channel TMS-compatible EEG system at a 5,000 Hz sampling rate, with the electrodes arranged as per the international 10–10 system. A figure-of-eight coil was used to deliver single TMS pulses over the left DLPFC at 120% of the individual’s resting motor threshold. About 150 trials were recorded for each assessment, and the interstimulus interval was randomly jittered between 4 and 6 s. Signal processing was carried out using EEGLAB and the TESA toolbox in MATLAB. EEG data were epoched from −1,000 to +1,000 msec around the TMS pulse. The immediate TMS artifact from −2 to +10 ms was removed and interpolated, down-sampled to 1,000 Hz, band-pass filtered (1–80 Hz), and notch filtered (50 Hz). The bad channels were determined by visual inspection and abnormal variance, and then interpolated. Ocular, muscle, decay and residual stimulation artifacts were removed by independent component analysis. Trials that were larger than 100 μV were discarded. Data were re-referenced to the common average and were baseline corrected with a − 500 to −50 ms prestimulus interval. The N100 and P200 amplitudes were computed in a time window of 80–140 and 150–250 ms, respectively, in the left-prefrontal electrode cluster (F1, F3, FC1 and FC3). When, after preprocessing, fewer than 100 artifact-free trials remained, the participant was excluded.

#### 2.7.2 fNIRS acquisition and preprocessing

The fNIRS data were collected with a continuous-wave system with a sampling rate of 10 Hz, at wavelengths of 760 nm and 850 nm. For the optode montage, 16 sources and 16 detectors were placed with an approximate distance of 30 mm between the source and detector, resulting in 48 measurement channels distributed over the bilateral prefrontal cortex. Left-DLPFC channels were determined based on the registered optode locations and their overlap with the F3 area of the international 10–20 system. The verbal-fluency paradigm consisted of three blocks of tasks with a 60 s duration each, with a 30 s resting baseline prior to each task block. Homer3 was used for pre-processing. Raw intensity data were transformed to OD, motion artifacts were corrected by wavelet filtering, data were band-pass filtered (0.01–0.20 Hz), and HBOD and OxyHb concentrations were calculated from the modified Beer–Lambert law. Scalp-coupling index values below 0.80, or a coefficient of variation (CV) greater than 15%, led to a channel being discarded. For the primary fNIRS outcome, the mean HbO2 response in the 5–25 s window after task onset in the *a priori*-defined channels over the left-DLPFC was considered.

#### 2.7.3 Resting-state fMRI acquisition and connectivity analysis

A 3-T MRI scanner was used to acquire resting-state fMRI data with a gradient-echo echo-planar imaging sequence. Typical acquisition parameters were repetition time 2,000 ms, echo time 30 ms, flip angle 90°, voxels 3 × 3 × 3 mm, 240 functional volumes and total scan time 8 min. The participants were asked to stay awake, with their eyes closed, and to try not to engage in structured thinking. Preprocessing was performed with SPM12 and the CONN toolbox. The first 10 volumes were removed, and slice-timing correction, rigid-body motion correction, coregistration with the anatomical image, normalization to Montreal Neurological Institute space, and spatial smoothing with a 6-mm full-width-at-half-maximum Gaussian kernel were then performed. Nuisance regression comprised 24 motion parameters and mean white-matter and cerebrospinal-fluid signals. Data were filtered in time between 0.01 and 0.08 Hz. It was declared that the volumes with framewise displacement > 0.50 mm were censored. The mean framewise displacement was calculated for each participant, and they were excluded if the value was greater than 0.30 mm or if more than 20% of their volumes had to be censored. The left-DLPFC seed was defined to be a 6 mm radius sphere centered at MNI coordinates *x* = −42, *y* = 36 and *z* = 30. Regions of the frontoparietal networks were identified based on the Yeo seven-network atlas. Pearson correlation coefficients were computed between the DLPFC seed and frontoparietal network time series, and Fisher’s r-to-z transformed.

### 2.8 Covariates and confounders

Demographic, clinical, injury-related, treatment-related, and site-level variables were pulled as potential covariates. Demographic variables such as sex and age were taken. Injury-related variables were time since injury, mechanism of injury, baseline injury severity, and baseline cognitive impairment. Clinical data consisted of the following: baseline FAB score, baseline MoCA executive domain score, baseline functional status, medication stability, and presence of any neurological or psychiatric comorbidities.

Rehabilitation-related covariates comprised rehabilitation type, frequency, duration and overall intensity within the observation window. The variables for the rTMS-exposed group, which were used in treatment, were as follows: stimulation frequency, stimulation intensity, pulses per session, completed sessions, cumulative stimulation dose and duration of treatment. The study site was added as a covariate since there were some variations between sites regarding clinical workflows, delivery of rehab, timing of assessment, and data acquisition procedures.

The participating center was included as a study-site covariate because rehabilitation delivery, assessment timing, and data acquisition protocols varied across institutions. These variables were selected *a priori* because they could influence both the likelihood of receiving rTMS and the probability of executive-function recovery, thereby acting as potential confounders in this observational cohort.

### 2.9 Statistical analysis

A retrospective cohort study design was used for statistical analysis. Because this retrospective cohort included all eligible records available during the predefined study period, no *a priori* sample-size calculation was performed. An approximate sensitivity analysis, assuming equal allocation and 22–24 participants per cohort, indicated that the available sample provided 80% power at a two-sided *α* level of 0.05 to detect standardized between-cohort differences of approximately Cohen’s d = 0.83–0.86. Therefore, the study was not adequately powered to detect small-to-moderate effects, particularly in the modality-specific biomarker and correlation analyses, which were considered exploratory. Continuous variables were expressed as either mean ± standard deviation or median (interquartile range), depending on distribution. Frequencies and percentages were used for categorical data. Standardized mean differences (SMDs) were calculated to quantify baseline balance between treatment groups. FAB, MoCA executive-domain, and TMT outcomes were analyzed using linear mixed-effects models with fixed effects for rTMS exposure, time, and the exposure-by-time interaction, and a participant-level random intercept. Models were adjusted for age, sex, baseline outcome score, injury severity, time since injury, rehabilitation intensity, medication stability, and study site. Admission GCS, PTA duration, DAI, and neurosurgical intervention were included in adjusted models when sufficiently complete; variables with substantial missingness or sparse categories were reported descriptively only. GOSE was analyzed using proportional-odds ordinal logistic regression. Biomarker changes were analyzed using multivariable linear regression with adjustment for the corresponding baseline biomarker value and the same prespecified covariates. Linearity, residual normality, homoscedasticity, proportional-odds assumptions, and multicollinearity were assessed; variance inflation factors below 5 were considered acceptable. Missing covariate data were handled using multiple imputation with 20 datasets, combined using Rubin’s rules. Continuous outcomes are reported as adjusted mean differences, GOSE as adjusted odds ratios, and all estimates with 95% confidence intervals. Exploratory biomarker comparisons were corrected using the Benjamini–Hochberg false-discovery-rate procedure, with FDR-adjusted q values <0.05 considered statistically significant. Propensity scores were estimated using logistic regression including age, sex, baseline executive-function score, time since injury, injury severity, rehabilitation intensity, medication stability, and study site. Stabilized inverse-probability-of-treatment weights were applied. Covariate balance was assessed using standardized mean differences, with an absolute SMD < 0.10 indicating acceptable balance. Weighted outcome models used robust standard errors.

Two-tailed testing was performed for all tests, and *p*-values <0.05 were considered statistically significant for primary clinical outcomes. Effect estimates were provided with 95% confidence intervals.

## 3 Results

### 3.1 Cohort identification and data availability

**Figure 1:**
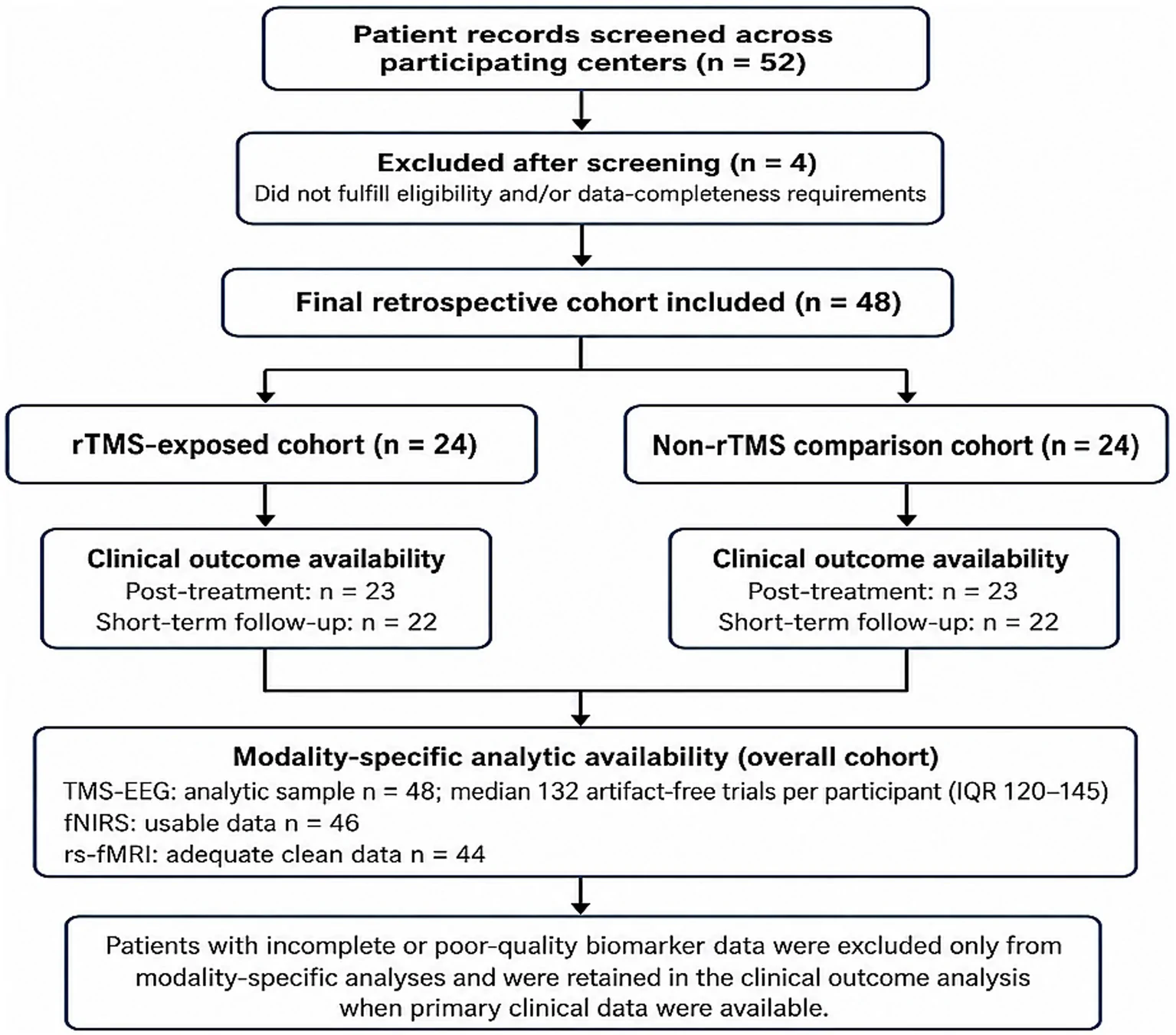
The retrospective cohort flow diagram.

Of the participating centers, a total of 52 patient records were screened. Forty-eight patients were included in the final retrospective cohort after excluding four records that did not meet eligibility or data-completeness criteria. Of the four excluded records, two lacked complete primary outcome data, one had uncertain rTMS exposure status, and one had insufficient documentation of rehabilitation intensity. Using documentation of treatment exposure, 24 patients were defined as exposed to rTMS and 24 patients were defined as non-rTMS comparison. The entire process of record screening, eligibility assessment, cohort classification, follow-up availability, and biomarker-specific data inclusion is illustrated in Figure 1.

At the early post-treatment assessment, clinical outcome data were available for 46 of 48 patients, with one patient from each cohort having incomplete post-treatment data. Clinical outcome data were available for 44 of 48 patients at short-term follow-up, with the remaining 4 patients (2 from each cohort) having incomplete documentation of follow-up. Median adherence to the rTMS protocol was 20 completed treatment sessions (19–20 sessions), indicating high treatment compliance among exposed participants.

The availability of multimodal biomarkers differed across the modalities. After quality-control procedures and preprocessing, usable TMS-EEG datasets were available for 48 participants, with a median of 132 accepted TMS-evoked trials per participant (IQR, 120–145 trials). FNIRS (fNIRS) datasets meeting predefined quality criteria were available for 46 participants, whereas resting-state functional MRI (rs-fMRI) data suitable for connectivity analyses were available for 44 participants. Patients with incomplete or poor-quality biomarker data were only excluded from the modality-specific analysis and included in the clinical outcome analysis if the primary clinical data were available.

### 3.2 Baseline demographic and clinical characteristics

There were no significant differences between the two cohorts at baseline. The mean age for the rTMS-exposed cohort was 39.8 ± 12.1 years, and for the non-rTMS comparison cohort was 39.4 ± 11.7 years. There were 7 female and 17 male patients in the rTMS-exposed cohort, and 8 female and 16 male patients in the non-rTMS comparison cohort. There were no significant differences in mean time since injury between cohorts (6.0 ± 2.7 months vs. 6.2 ± 2.9 months), Table 1.

**Table 1:** Baseline demographic, injury-related, clinical, and rehabilitation characteristics.

Characteristic	rTMS-exposed (*n* = 24)	Non-rTMS comparison (*n* = 24)	Unweighted SMD	Weighted SMD*
Age, years	39.8 ± 12.1	39.4 ± 11.7	0.034	0.021
Female sex, *n* (%)	7 (29.2)	8 (33.3)	−0.090	−0.028
Time since injury, months	6.0 ± 2.7	6.2 ± 2.9	−0.071	−0.031
Road-traffic injury, *n* (%)	15 (62.5)	14 (58.3)	0.085	0.036
Admission GCS, mean ± SD†	9.8 ± 3.1	10.1 ± 3.0	−0.098	−0.041
Mild TBI, *n* (%)†	6 (25.0)	7 (29.2)	−0.094	−0.036
Moderate TBI, *n* (%) †	10 (41.7)	9 (37.5)	0.085	0.029
Severe TBI, *n* (%) †	8 (33.3)	8 (33.3)	0	0
PTA duration, days, median (IQR) †	8 (4–15)	7 (3–14)	—	—
Frontal lesion involvement, *n* (%) †	10 (41.7)	9 (37.5)	0.085	0.032
Diffuse axonal injury, *n* (%) †	8 (33.3)	7 (29.2)	0.09	0.038
Abnormal structural imaging, *n* (%) †	20 (83.3)	19 (79.2)	0.107	0.045
Previous neurosurgical intervention, *n* (%) †	6 (25.0)	5 (20.8)	0.099	0.04
FAB score	12.8 ± 2.1	12.6 ± 2.2	0.093	0.044
MoCA executive-domain score	3.3 ± 1.1	3.2 ± 1.2	0.087	0.038
TMT-A, seconds	49.3 ± 15.8	50.7 ± 16.1	−0.088	−0.043
TMT-B, seconds	118.6 ± 36.9	121.8 ± 39.1	−0.084	−0.047
GOSE, median (IQR)	5 (4–6)	5 (4–6)	—	—
Rehabilitation sessions/week	3.0 ± 1.1	3.1 ± 1.0	−0.095	−0.070

Baseline measures of executive function were also similar. Injury-related characteristics were also broadly comparable between cohorts, including admission GCS, injury-severity category, PTA duration, lesion distribution, DAI, structural neuroimaging abnormalities, and previous neurosurgical intervention (Table 1). The mean (FAB) score was 12.8 ± 2.1 in the rTMS-exposed cohort and 12.6 ± 2.2 in the comparison (non-rTMS) cohort. The mean MoCA executive-domain score was 3.3 ± 1.1 and 3.2 ± 1.2, respectively. Baseline TMT A and B scores, Glasgow Outcome Scale–Extended score, and exposure to rehab were comparable across cohorts, justifying subsequent adjusted longitudinal analyses. These findings suggest that the two cohorts were broadly similar at baseline with respect to measured demographic and clinical characteristics. Nevertheless, because treatment allocation was not randomized, the possibility of residual confounding from unmeasured variables cannot be excluded.

#### 3.2.1 Baseline balance assessment

Before weighting, the largest absolute SMD was 0.107. After weighting, all measured covariates achieved acceptable balance, with a maximum absolute SMD of 0.070. Stabilized weights ranged from 0.74 to 1.39, and the effective sample size was 45.6. Visual inspection of the propensity-score distributions demonstrated substantial overlap between the rTMS-exposed and non-rTMS comparison cohorts, indicating adequate common support and supporting the comparability of the two study cohorts (Supplementary Figure S1).

### 3.3 rTMS exposure and rehabilitation characteristics

Adjusted longitudinal analyses using multivariable regression models demonstrated findings consistent with the unadjusted analyses. After adjustment for age, sex, baseline executive-function score, time since injury, rehabilitation intensity, medication stability, study site, and available injury severity variables, the association between documented rTMS exposure and executive-function outcomes remained statistically significant. In the weighted analysis, rTMS exposure remained associated with greater improvement in FAB score (adjusted difference, 3.0 points; 95% CI, 1.4–4.5; *p* < 0.001) and MoCA executive-domain score (adjusted difference, 2.7 points; 95% CI, 1.2–4.2; *p* = 0.001). Exposure to treatment in the rTMS group is presented in Table 2. Records of treatment showed high-frequency rTMS to the left DLPFC. Stimulation parameters were: 10 Hz stimulation, 2,500 pulses per session, and intensity delivered at 120% of resting motor threshold. Sessions were scheduled five times a week for 4 weeks (20 planned sessions and 50,000 planned pulses of stimulation).

**Table 2:** Exposure and rehabilitation properties of rTMS.

Domain	Variable	Value
rTMS treatment exposure	Stimulation target	Left DLPFC
rTMS treatment exposure	Stimulation frequency	10 Hz
rTMS treatment exposure	Pulses per session	2,500
rTMS treatment exposure	Stimulation intensity	120% RMT
rTMS treatment exposure	Treatment schedule	5 sessions/week for 4 weeks
rTMS treatment exposure	Planned total sessions	20
rTMS treatment exposure	Planned cumulative stimulation dose	50,000 pulses
rTMS adherence	Completed sessions, median (IQR)	20 (19–20)
Rehabilitation exposure	rTMS-exposed cohort, sessions/week, mean ± SD	3.0 ± 1.1
Rehabilitation exposure	Non-rTMS comparison cohort, sessions/week, mean ± SD	3.1 ± 1.0

Good compliance with the treatment schedule is documented. The median number of rTMS sessions completed was 20, with a range of 19–20 sessions. The intensity of conventional rehabilitation was similar between the two cohorts, averaging 3.0 ± 1.1 sessions/week in the rTMS-exposed cohort and 3.1 ± 1.0 sessions/week in the non-rTMS comparison cohort, indicating comparable background rehabilitation exposure.

### 3.4 Executive function primary clinical outcomes

The results for primary executive-function outcomes are reported in Table 3. rTMS exposure was linked to larger improvement in executive function measures in adjusted longitudinal analyses compared with the non-rTMS comparison cohort. For both FAB and MoCA executive-domain scores, the exposure-by-time interaction favored the rTMS-exposed cohort.

**Table 3:** Primary executive-function outcomes.

Outcome	Timepoint	rTMS-exposed cohort	Non-rTMS comparison cohort	Adjusted between-cohort difference	95% CI	*p*-value
FAB score	Baseline, mean ± SD	12.8 ± 2.1	12.6 ± 2.2	—	—	0.72
FAB score	Post-treatment mean change	3.7	0.6	3.1	1.6 to 4.6	<0.001
FAB score	Short-term follow-up mean change	3.3	0.6	2.7	1.2 to 4.1	0.001
MoCA executive-domain score	Baseline, mean ± SD	3.3 ± 1.1	3.2 ± 1.2	—	—	0.84
MoCA executive-domain score	Post-treatment mean change	3.1	0.3	2.8	1.3 to 4.3	0.001
MoCA executive-domain score	Short-term follow-up mean change	2.7	0.4	2.3	0.9 to 3.7	0.002

The rTMS-exposed cohort had an increase of +3.7 points at post-treatment assessment on the FAB, while the non-rTMS comparison cohort had a difference of +0.6 points. The adjusted between-cohort difference was +3.1 points (95% CI, 1.6 to 4.6; *p* < 0.001). The rTMS-exposed group continued to show improvement at short-term follow-up (+3.3 points) vs. the non-rTMS comparison group (+0.6 points), representing an adjusted between-cohort difference of +2.7 points (95% CI, 1.2 to 4.1; *p* = 0.001).

On the MoCA executive-domain score, the rTMS-exposed cohort showed a greater increase of +3.1 points at post-treatment assessment relative to +0.3 points in the non-rTMS comparison cohort. The adjusted between-cohort difference was +2.8 points (95% CI, 1.3 to 4.3; p = 0.001). The rTMS cohort continued to improve by +2.7 points at short-term follow-up, whereas the comparison cohort improved by +0.4 points, for an adjusted difference of +2.3 points (95% CI, 0.9 to 3.7; *p* = 0.002).

### 3.5 Secondary clinical and functional outcomes

Secondary clinical and functional outcomes are included in Table 4. Compared with the non-rTMS comparison cohort, functional recovery assessed using the Glasgow Outcome Scale–Extended was more favorable in the rTMS-exposed cohort at both post-treatment assessment and short-term follow-up. The odds of a better GOSE category at post-treatment assessment were greater with rTMS exposure (adjusted OR, 2.2; 95% CI, 1.2 to 4.0; *p* = 0.010). This association was also seen at short-term follow-up with an adjusted odds ratio of 2.0 (95% CI, 1.1 to 3.8; *p* = 0.020). In the rTMS-exposed group, processing speed and cognitive flexibility also increased. The mean change from baseline in Trail-Making Test A was −9.5 s in the rTMS-exposed cohort and −1.6 s in the non-rTMS comparison cohort, with an adjusted between-cohort difference of −7.9 s (95% CI, −12.3 to −3.6; *p* < 0.001). There was a mean change of −23.7 s and −5.2 s, respectively, for Trail-Making Test B, with a corrected between-cohort change of −18.5 s (95% CI, −30.2 to −6.7; *p* = 0.003). Difficulty ratings on the self-report measure of cognitive functioning also favored the rTMS-exposed group, revealing a between-group difference of −0.6 SD units at the post-treatment assessment (*p* = 0.010).

**Table 4:** Secondary clinical and functional outcomes.

Outcome	Timepoint	rTMS-exposed cohort	Non-rTMS comparison cohort	Adjusted estimate	95% CI	*p*-value
GOSE	Post-treatment	NR	NR	OR 2.2	1.2 to 4.0	0.01
GOSE	Short-term follow-up	NR	NR	OR 2.0	1.1 to 3.8	0.02
TMT-A, seconds	Post-treatment mean change	−9.5	−1.6	−7.9	−12.3 to −3.6	<0.001
TMT-B, seconds	Post-treatment mean change	−23.7	−5.2	−18.5	−30.2 to −6.7	0.003
Patient-reported cognition	Post-treatment	NR	NR	−0.6 SD units	NR	0.01

### 3.6 Biomarker data availability and quality (multimodal)

The availability and quality of multimodal biomarker data are summarized in Table 5. For each biomarker analyzed, the number of participants varied by modality, as modality-specific quality-control procedures and data-completeness criteria were used prior to analysis. If a participant did not have complete or high-quality biomarker data in a specific modality, they were excluded from analyses only for that modality. If full clinical data were available, they were included in the primary clinical outcome analyses.

**Table 5:** Access to and quality of multimodal biomarker data.

Biomarker modality	Records reviewed	Usable data after quality control, *n* (%)	Excluded or unavailable data, *n* (%)	Final analytic sample
TMS-EEG	48	48 (100.0)	0 (0.0)	48
fNIRS	48	46 (95.8)	2 (4.2)	46
Resting-state fMRI	48	44 (91.7)	4 (8.3)	44

Two fNIRS datasets were discarded as less than half of the fNIRS channels in the predefined left-DLPFC were deemed to be of good signal quality. Four resting-state fMRI datasets did not pass quality control. They were excluded due to high levels of head motion (mean framewise displacement >0.30 mm) and/or a high number of censored functional volumes (data from more than 20% of volumes). Exclusion criteria: No TMS-EEG data sets were excluded, as all participants had 100 or more artifact-free trials remaining after data pre-processing.

After the standardized preprocessing and quality-control procedures, usable TMS-EEG recordings were obtained for the analytic cohort, with a median of 132 artifact-free trials per participant (interquartile range [IQR] 120–145 trials). After image-quality assessment and motion-control procedures, 46/48 participants had datasets of good quality for fNIRS (fNIRS) and 44/48 had datasets of good quality for resting-state functional magnetic resonance imaging (rs-fMRI) suitable for connectivity analyses.

The decreased number of participants available for the fNIRS and rs-fMRI analyses is due to the retrospective nature of the study and variations in availability of clinical data across participating centers. Therefore, results of the multimodal biomarker analyses should be viewed as exploratory and appropriately interpreted, as the study was not specifically designed to assess differences in neurophysiological or neuroimaging biomarkers.

### 3.7 Neurophysiological and imaging-response to rTMS exposure

The multimodal changes in the neurophysiological and imaging domains are summarized in Table 6. The rTMS-exposed cohort had larger changes in electrophysiological, hemodynamic, and network measures of frontal target engagement compared with the non-rTMS comparison cohort. Such results suggest that clinical executive change was linked to rTMS exposure as well as to quantifiable modulation of frontal cortical and frontoparietal networks.

**Table 6:** Across multiple modalities, changes in biomarkers related to exposure to rTMS were observed.

Modality	Biomarker	rTMS-exposed cohort change	Non-rTMS comparison cohort change	Adjusted between-cohort difference	95% CI	FDR-*q* value
TMS-EEG	N100 amplitude, μV	−3.8	−0.6	−3.2	−4.8 to −1.6	0.006
TMS-EEG	P200 amplitude, μV	2.6	0.5	2.1	0.3 to 3.8	0.048
fNIRS	Left DLPFC HbO, μmol/L	0.24	0.03	0.21	0.09 to 0.33	0.01
rs-fMRI	DLPFC–FPN connectivity, Fisher-z	0.2	0.02	0.18	0.07 to 0.29	0.02

The rTMS-exposed group exhibited a greater increase in magnitude of the N100 over frontal regions (−3.2 μV (95% CI, −4.8 to −1.6); FDR-*q* = 0.006). Similarly, an increase in P200 amplitude was observed in the rTMS-exposed group (+2.1 μV (95% CI: 0.3–3.8), FDR-*q* = 0.048). Task-evoked HbO2 response over the left DLPFC was greater in the rTMS-exposed cohort compared to the other group (+0.21 μmol/L, 95% CI: 0.09 to 0.33 μmol/L, FDR-q = 0.010). A greater change in DLPFC – frontoparietal network connectivity was seen in the rTMS-exposed cohort, with a between-cohort difference of +0.18 Fisher-z units (95% CI, 0.07 to 0.29; FDR-*q* = 0.020).

### 3.8 Relationship between biomarker changes and executive-function recovery

**Figure 2:**
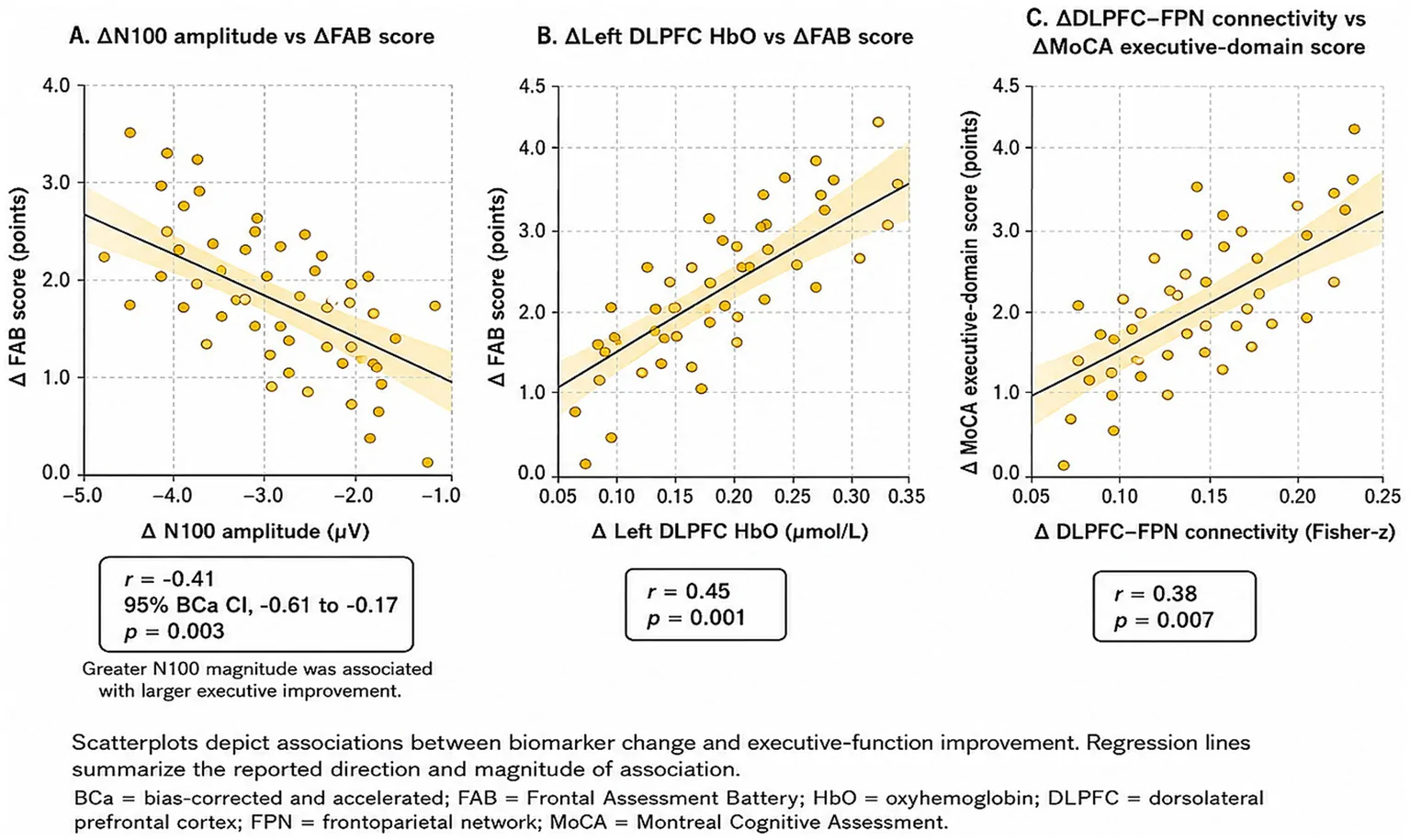
Biomarker–clinical coupling. Scatterplots show associations between (A) ΔN100 amplitude and ΔFAB score, (B) ΔLeft DLPFC HbO and ΔFAB score, and (C) ΔDLPFC–FPN connectivity and ΔMoCA executive-domain score. Regression lines indicate the direction of association. FAB = Frontal Assessment Battery; HbO = oxyhaemoglobin; DLPFC = dorsolateral prefrontal cortex; FPN = frontoparietal network; MoCA = Montreal Cognitive Assessment.

Relationships between changes in biomarkers and changes in executive function are summarised in Table 7 and depicted in Figure 2. These studies investigated whether there were neurophysiological and imaging changes that were correlated with improved executive outcome. In general, higher frontal cortical reactivity, higher task-related prefrontal activation and higher DLPFC-frontoparietal connectivity correlated with larger executive-function gains.

**Table 7:** Correlation of changes in biomarkers and executive-function gains.

Biomarker predictor	Clinical outcome	Correlation coefficient	95% CI	*p*-value
ΔN100 amplitude	ΔFAB score	*r* = −0.41	−0.61 to −0.17	0.003
ΔN100 amplitude	ΔMoCA executive-domain score	*r* = −0.38	NR	0.006
ΔLeft DLPFC HbO	ΔFAB score	*r* = 0.45	NR	0.001
ΔDLPFC–FPN connectivity	ΔMoCA executive-domain score	*r* = 0.38	NR	0.007

A change in N100 amplitude over the predefined left-prefrontal electrode cluster comprising F1, F3, FC1, and FC3 was correlated with improvement in (FAB) score (*r* = −0.41, 95% BCa CI, −0.61 to −0.17; *p* = 0.003). The negative direction shows that a higher magnitude of N100 was linked to greater executive improvement. Change in N100 amplitude over the same left-prefrontal electrode cluster was also correlated with improvement in the MoCA executive-domain score (*r* = −0.38, *p* = 0.006). In the fNIRS data, the increases in left DLPFC HbO2 response correlated with greater increases in FAB score (*r* = 0.45, *p* = 0.001). The change in resting-state fMRI connectivity between DLPFC and frontoparietal network positively correlated with improvement in MoCA executive-domain score (*r* = 0.38; *p* = 0.007).

### 3.9 Sensitivity analyses

The results of sensitivity analyses are presented in Table 8. The direction and magnitude of the associations between documented rTMS exposure and executive-function outcomes were generally consistent across alternative analytic specifications. Complete-case analyses yielded estimates similar to the primary analysis. Analyses restricted to participants with high treatment adherence showed comparable associations, supporting the robustness of the exposure–outcome findings.

**Table 8:** Sensitivity analyses for primary EF outcomes.

Sensitivity analysis	Outcome domain	Result summary	Statistical finding	Interpretation
Complete-case analysis	FAB and MoCA executive-domain score	Findings agreed with the primary analysis	Directionally consistent	Missing outcome data did not materially alter the primary findings
High-adherence analysis	FAB and MoCA executive-domain score	Associations were similar in direction and magnitude	Directionally consistent	Results were robust among patients completing the treatment/observation window.
Time-since-injury strata	Executive-function outcomes	No evidence of differential association by injury timing	Interaction *p* = 0.42	The observed association was not restricted to a single recovery-window subgroup.
Rehabilitation intensity interaction	Executive-function outcomes	No meaningful effect modification	Interaction *p* > 0.20	Differences in rehabilitation exposure did not explain the findings
Stable medication-use interaction	Executive-function outcomes	No meaningful effect modification	Interaction *p* > 0.20	Findings were not materially modified by medication stability

There was no apparent heterogeneity in the observed associations by time since injury in the exploratory effect-modification analysis. The treatment exposure by time-since-injury strata interaction was not statistically significant (*p* = 0.42). Stable use of medications or intensity of rehabilitation did not materially change the observed exposure-associated estimates, as interaction tests were not significant (all interaction *p* > 0.20). These findings indicate that the main results do not depend on important clinically relevant sensitivity checks and were not due to a substantial imbalance in rehabilitation exposure or medication stability.

## 4 Discussion

High-frequency rTMS applied to the left DLPFC was associated with greater executive-function recovery in this retrospective multicenter observational cohort study. The rTMS-exposed cohort demonstrated more improvement on FAB and MoCA executive-domain scores at post-treatment assessment that remained at short-term follow-up compared to the non-rTMS comparison cohort. These findings were supported by favorable secondary outcomes, including improved GOSE category and better Trail Making Test A and B performance. In addition, the rTMS-exposed cohort demonstrated changes in multimodal biomarkers, including TMS-EEG N100/P200 responses, increased task-evoked left DLPFC HbO2 responses measured by fNIRS, and stronger DLPFC–frontoparietal functional connectivity on resting-state fMRI. Collectively, these observations suggest an association between documented rTMS exposure, executive-function outcomes, and frontal-network physiological measures. However, because of the retrospective observational design and lack of randomization, these findings should not be interpreted as evidence of a causal treatment effect (Franke et al., 2022; Aleid et al., 2025).

The observed clinical and biomarker findings support the hypothesis that repeated prefrontal stimulation may be associated with modulation of local cortical activity and distributed frontoparietal executive-control networks. Nevertheless, the present study cannot establish the biological mechanisms underlying these associations. The inclusion of three complementary biomarker modalities represents an important strength because previous neuromodulation studies in TBI have often relied on either clinical outcomes or physiological measures alone. By integrating electrophysiological, hemodynamic, and functional connectivity measures, the present findings provide converging evidence that may help characterize neurophysiological changes accompanying executive-function recovery following documented rTMS exposure. These multimodal observations should therefore be considered hypothesis-generating rather than definitive evidence of a stepwise biological mechanism (Lindsey et al., 2023; Beynel et al., 2020; He et al., 2025).

The observed relationships between executive-function outcomes and reported exposures to rTMS are broadly consistent with prior research indicating potential for high-frequency prefrontal rTMS in select patients with TBI. However, the evidence is mixed. While objective cognitive improvements have been limited, high-frequency prefrontal rTMS has previously been linked to improvements in post-concussive symptoms, neurobehavioral outcomes and EEG measures. The present cohort showed a larger increase in standardized executive-function measures than these studies. This does not necessarily indicate a difference in treatment effectiveness, but could be due to a difference in patient selection, stimulation protocol, time since injury, rehabilitation intensity or outcome measures. A combination of frontal executive-function assessments and multimodal biomarkers might have enhanced clinically relevant association detection. But this interpretation needs to be confirmed in prospective randomized studies (Franke et al., 2022).

Unlike the randomized trial of high-frequency left-DLPFC rTMS for cognition in chronic diffuse axonal injury (DAI), there was a relationship between documented rTMS exposure and executive-function outcomes in the present observational cohort. These conflicting results can be attributed to a number of factors such as variation in patient populations, injury characteristics, timing of intervention, intensity of rehabilitation, and residual integrity of the network. Patients enrolled in the current study were assessed 3 to 12 months post-injury, a time when a significant amount of neurological recovery and neuroplasticity may also occur spontaneously, thus playing a major role in cognitive recovery. Thus, it is not possible to ascertain the relative contribution of natural recovery, the multidisciplinary rehabilitation and the documented exposure to rTMS to the clinical changes observed. These results thus suggest that future, carefully designed prospective randomized studies, which are able to distinguish treatment effects from spontaneous recovery and take patient selection, target engagement, and injury heterogeneity into account, are important (Neville et al., 2019).

Recent systematic reviews have highlighted the potential promise of rTMS in TBI, but also the heterogeneity of the studies, with varying stimulation sites, frequencies, treatment durations, TBI phenologies, and cognitive outcomes. The current study aims to tackle some of these issues by employing a specific method of L-DLPFC stimulation and domain-specific executive measures. The current investigation ads further observationally derived evidence by investigating documented rTMS exposure in the context of executive-function measures across several domains and additional biomarker measures in complementary modalities. As such, the current results should be viewed as evidence of the feasibility of combining clinical and physiological outcome measures in future studies but not as proof of the effectiveness of rTMS or as a proof of concept for a biomarker-based rehabilitation strategy. However, further prospective, randomized controlled trials with standardized protocols are required to assess if these multimodal biomarkers have clinically relevant information with regard to treatment responsiveness (Lindsey et al., 2023; Nousia et al., 2022).

Improvements were seen in the FAB and MoCA executive domain scores, and the assessment of cognitive processes, including planning, working memory, and cognitive flexibility, should be carried out. The findings, however, were likely due to the multidisciplinary neurorehabilitation received by all subjects, as patients were assessed during the 3–12 months after injury. The observed improvements may also have been influenced by spontaneous neurological recovery, intensity of rehabilitation, and other clinical factors that occurred during the ongoing period of spontaneous neurological recovery (Lindsey et al., 2023; Nousia et al., 2022).

Changes in the FAB and MoCA executive-domain scores were larger in the rTMS-exposed cohort and remained stable at short-term follow-up, and correlated with biomarker changes. The information presented in these findings, however, only suggests an exposure–outcome relationship and does not prove rTMS increased the efficacy of executive recovery or added to rehabilitation. The observed differences could also be due to spontaneous recovery, treatment-selection factors, or residual confounding (Calderone et al., 2024).

The use of several complementary modalities of neurophysiology and neuroimaging in the present study is an important asset for assessing the change in the cortex and network of the brain associated with documented rTMS exposure. Biomarker data, however, were only available for a subsample of participants, so these analyses should be considered exploratory. The results of this study showed that the amplitude of the TMS-EEG N100 and P200 responses was increased after rTMS exposure, indicating that there was a change in the physiological response of the cortex to this stimulus. While the N100 component is often thought to reflect GABA<sub > B</sub > −mediated inhibitory processes in the cortex, there is a growing body of evidence suggesting that a variety of physiological processes such as sensory evoked responses, auditory processing, somatosensory inputs, attention and general cortical network activity are all relevant to the N100. As such, alterations in N100 cannot be considered a specific or exclusive sign of cortical inhibition. The correlation between N100 modulation and executive-function improvement observed here is consistent with previous studies that have suggested that TMS-EEG measures could be used as candidate biomarkers of the responsiveness of the cortex. Still, these are exploratory and need to be confirmed in larger, prospective studies with standardized acquisition and preprocessing protocols (Prokop-Millar et al., 2025; Premoli et al., 2014).

These results from fNIRS add to the evidence of the relationship between reported exposure to rTMS and prefrontal activation during task performance. This increased HbO response in the left DLPFC during the verbal-fluency task, combined with the positive correlation between prefrontal activation and FAB improvement, suggests that greater prefrontal activation may be related to improvements in executive function. Due to the observational design and the number of participants who provided fNIRS data, these results should be viewed with care. The greater task-related HbO responses may be attributable to multiple mechanisms, such as spontaneous neuroplasticity, ongoing rehabilitation, compensatory recruitment of cortex, and/or individual differences in recovery and not necessarily a pure physiological action of rTMS. The findings are in line with the previous rehabilitation studies that showed fNIRS as a tool to measure the changes in the hemodynamic activity of the cortex in the course of neuro-modulation and cognitive rehabilitation; however, whether fNIRS is useful as a predictive biomarker for treatment response in future longitudinal studies remains to be confirmed (Zhang et al., 2024; Curtin et al., 2019).

The rTMS-exposed group exhibited greater DLPFC–frontoparietal connectivity in resting-state fMRI and greater MoCA executive-domain improvement, these changes being correlated. This finding can be interpreted in the context of other network-based theories of rTMS, which postulate that the stimulation effects are not confined to the target cortex but spread out via functional networks. After a systematic review of rTMS studies using resting-state fMRI, it has been reported that rTMS can produce measurable changes in connectivity both within and beyond the stimulated network. The present findings extend these observations by demonstrating similar associations in a retrospective TBI cohort; however, confirmation in adequately powered prospective studies is required before these biomarkers can be considered reliable indicators of treatment response (Fox et al., 2012; Eldaief et al., 2011; Cocchi et al., 2015).

Results from TMS-EEG, fNIRS and resting-state fMRI enable us to propose a hypothesis about the possible order of neurophysiological changes during recovery of executive functions. In particular, changes in TMS-EEG responses, increases in activation during task in prefrontal areas and enhanced frontoparietal connectivity might be complementary physiological measures that accompany clinical improvements. However, there is no current evidence that supports a biological sequence from increases in cortical excitability to hemodynamic activation to network reorganization to behavioral recovery. All biomarker analysis was correlational and observational, and therefore causality between these measures cannot be drawn. Taken together, these multimodal biomarker findings should be interpreted as exploratory indicators of target engagement rather than definitive evidence of the biological mechanisms underlying executive-function recovery. Although the observed associations are biologically plausible, the present observational study was not designed to establish mechanistic pathways or causal relationships. Accordingly, these findings should be considered hypothesis-generating and require validation in adequately powered prospective studies incorporating standardized biomarker acquisition and longitudinal assessment (Premoli et al., 2014; Mihara and Miyai, 2016).

The multimodal findings may also help explain why previous TBI neuromodulation studies have reported inconsistent clinical outcomes. Rather than indicating that unsuccessful studies failed to engage the intended cortical target, differences across studies may reflect variability in patient characteristics, injury severity, stimulation parameters, rehabilitation intensity, outcome measures, and timing after injury. The results from the present study indicate that the use of stimulation and imaging together with neurophysiologic readouts can help better understand treatment response and aid in more appropriate patient selection. Nevertheless, these observations remain exploratory and should be interpreted as generating hypotheses for future biomarker-guided clinical trials rather than establishing validated biomarkers for patient selection (Fox et al., 2012; Cash et al., 2019; Bergmann and Hartwigsen, 2021).

The present findings support further prospective evaluation of high-frequency left-DLPFC rTMS in patients with persistent executive dysfunction following TBI. The observed associations between documented rTMS exposure, executive-function outcomes, processing speed, functional recovery, and multimodal physiological measures support further investigation of this approach. However, because treatment allocation was not randomized and residual confounding cannot be excluded, these findings should not be interpreted as evidence of clinical efficacy. Instead, they support the feasibility of conducting future randomized controlled trials to determine whether adjunctive rTMS provides meaningful clinical benefit beyond standard neurorehabilitation alone (Bergquist, 2009; Polanía et al., 2018).

Additional safety findings support the clinical feasibility of this approach. Adverse events, if any, were mild and self-limited, and no seizures or serious adverse events were reported in either cohort. Although these observations support the feasibility of administering rTMS within routine neurorehabilitation practice, the relatively small sample size limits the ability to detect uncommon adverse events. Larger prospective studies with systematic safety monitoring are therefore required (Rossi et al., 2009).

Similarly, the multimodal biomarker approach adopted in this study could provide insights into the potential utility of combining TMS-EEG, fNIRS, and resting-state fMRI to capture changes in the physiology of the cortex and networks underlying executive-function recovery. The current study, however, does not validate these biomarkers for use in clinical decision-making. The possible application to biomarker-guided neurorehabilitation is preliminary and needs validation by standardized acquisition procedures, validation by external groups, and prospective multicenter studies (Mihara and Miyai, 2016; Chen et al., 2025).

A number of limitations should be recognized. First, the retrospective, observational nature of the study does not allow causal inference, and residual confounding, treatment-selection bias, and confounding by indication cannot be completely excluded despite adjustment for measured covariates. In routine clinical practice, patients selected to receive rTMS may also differ from those receiving standard neurorehabilitation in factors that are difficult to measure, such as rehabilitation adherence, individual motivation, treatment preferences, clinician selection, or other unrecorded clinical considerations. These unmeasured factors may have influenced both treatment allocation and recovery outcomes and should therefore be considered when interpreting the observed associations. Secondly, the relatively small sample size reduced the statistical power and the potential for Type I and II error, especially in the modality-specific biomarker analyses. As such, the biomarker results are exploratory and not conclusive. Third, important clinical variables such as injury severity, lesion characteristics, and other structural MRI findings were not available in all participants. Thus, we were not able to do more extensive adjusted analyses. Fourth, not all patients had patient-level biomarker data available for all modalities, and only patients with usable TMS-EEG, fNIRS, or resting-state fMRI datasets were used in modality-specific analyses, potentially introducing selection bias. Fifth, participants were assessed within 3 to 12 months following injury, a time when spontaneous neurological recovery and neurological changes due to rehabilitation and learning may significantly impact their cognitive and physiological measurements. Thus, it is not possible to conclude the relative contribution of rTMS exposure to the observed changes. Last, only short-term follow-up was performed, and the durability of the clinical and biomarker changes is unknown. Therefore, the current results should be considered preliminary and hypothesis-generating until the results are replicated in larger, prospective, randomized controlled trials (Concato et al., 2017).

Future research should focus on well-powered, prospective multicenter RCTs with uniform stimulation protocols, thorough baseline phenotyping, uniform collection of biomarkers, and longer follow-up. Formal mediation analyses will be possible with detailed clinical, neuroimaging and neurophysiological data collected systematically, which will help to distinguish whether the biomarker change is mechanistically related to clinical recovery, or whether it happens along with clinical recovery. Future studies should also include comparisons of MRI-guided neuronavigation, Beam-F3 localization, and connectivity-guided targeting of these approaches to ascertain if individualized stimulation could enhance network engagement and clinical outcome. Also, in larger studies, it is important to explore whether early changes in TMS-EEG, fNIRS or resting-state fMRI could accurately predict the long-term recovery of executive functions and inform individualized treatment approaches. Multimodal biomarkers should be considered as research instruments until valid evidence is provided that represents the biomarker approach to neurorehabilitation. Lastly, trials in the future should examine rTMS in tandem with structured executive-function rehabilitation, to see if combined neuromodulation and cognitive training results in lasting functional gains over conventional rehabilitation alone.

## 5 Conclusion

The present retrospective multicenter observational cohort study found that outcomes for executive-function measures after TBI were greater with documented exposure to high-frequency rTMS applied to the left DLPFC as compared to a non-rTMS rehabilitation cohort. This included greater improvement in executive domain scores on the FAB and MoCA along with improved functional recovery and Trail Making Test performance over the course of the observation period in the rTMS-exposed cohort.

Exploratory multimodal biomarker analyses revealed parallel changes in TMS-EEG N100/P200 responses, greater task-related fNIRS HbO activation in the left DLPFC, and enhanced functional connectivity between the DLPFC and frontoparietal areas on resting-state fMRI. The observed design of the study and small sample size of the biomarkers do not allow any conclusions to be drawn regarding causal mechanisms and the validation of a biomarker-guided neurorehabilitation framework for rTMS exposure and measurable neurophysiological and neuroimaging changes.

High-frequency left-DLPFC rTMS could be a new tool to add to multidisciplinary neurorehabilitation for specific TBI patients with ongoing executive dysfunction. The present findings should be interpreted as demonstrating associations; however, as treatment allocation was not randomized and residual confounding cannot be excluded, the results should not be considered as evidence of treatment efficacy.

These results suggest that high-frequency left-DLPFC rTMS will be a subject of future RCTs. It is important to note that treatment allocation was not random and that residual confounding cannot be ruled out, which means that the present findings indicate associations and not the efficacy of treatment or clinical benefit.

## Data Availability

The raw data supporting the conclusions of this article will be made available by the authors, without undue reservation.

## References

[ref1] Aleid A. M. AlShammri M. S. Aldanyowi S. N. Alessa A. A. Alhussain A. A. Al Mutair A. (2025). Use of repetitive transcranial magnetic stimulation in traumatic brain injury: a systematic review and meta-analysis of randomized controlled trials. Surg. Neurol. Int. 16:175. doi: 10.25259/SNI_926_2024, 40469336 PMC12134812

[ref2] Bergmann T. O. Hartwigsen G. (2021). Inferring causality from noninvasive brain stimulation in cognitive neuroscience. J. Cogn. Neurosci. 33, 195–225. doi: 10.1162/jocn_a_01591, 32530381

[ref3] Bergquist T. (2009). Evidence-based cognitive rehabilitation: systematic review of the literature from 2009 through 2014. Arch. Phys. Med. Rehabil. 100, 1515–1533. doi: 10.1016/j.apmr.2019.02.011, 30926291

[ref4] Beynel L. Powers J. P. Appelbaum L. G. (2020). Effects of repetitive transcranial magnetic stimulation on resting-state connectivity: a systematic review. NeuroImage 211:116596. doi: 10.1016/j.neuroimage.2020.116596, 32014552 PMC7571509

[ref5] Bouchard A. E. Renauld E. Fecteau S. (2023). Changes in resting-state functional MRI connectivity during and after transcranial direct current stimulation in healthy adults. Front. Hum. Neurosci. 17:1229618. doi: 10.3389/fnhum.2023.1229618, 37545594 PMC10398567

[ref6] Calderone A. Cardile D. Gangemi A. De Luca R. Quartarone A. Corallo F. . (2024). Traumatic brain injury and neuromodulation techniques in rehabilitation: a scoping review. Biomedicine 12:438. doi: 10.3390/biomedicines12020438, 38398040 PMC10886871

[ref7] Cash R. F. Zalesky A. Thomson R. H. Tian Y. Cocchi L. Fitzgerald P. B. (2019). Subgenual functional connectivity predicts antidepressant treatment response to transcranial magnetic stimulation: independent validation and evaluation of personalization. Biol. Psychiatry 86, e5–e7. doi: 10.1016/j.biopsych.2018.12.002, 30670304

[ref8] Chen D. Shi J. Tao B. Zhao X. Zhao Z. Li S. . (2025). A novel transfer learning-based hybrid EEG-fNIRS brain-computer Interface for intracerebral Hemorrhage rehabilitation. Adv. Sci. 12:e05426. doi: 10.1002/advs.202505426, 40831229 PMC12631896

[ref9] Cocchi L. Sale M. V. Lord A. Zalesky A. Breakspear M. Mattingley J. B. (2015). Dissociable effects of local inhibitory and excitatory theta-burst stimulation on large-scale brain dynamics. J. Neurophysiol. 113, 3375–3385. doi: 10.1152/jn.00850.2014, 25717162 PMC4443609

[ref10] Concato J. Shah N. Horwitz R. I. (2017). “Randomized, controlled trials, observational studies, and the hierarchy of research designs,” in Research Ethics, (Milton Park: Routledge). 10.1056/NEJM200006223422507 PMC1557642 10861325

[ref11] Curtin A. Tong S. Sun J. Wang J. Onaral B. Ayaz H. (2019). A systematic review of integrated functional near-infrared spectroscopy (fNIRS) and transcranial magnetic stimulation (TMS) studies. Front. Neurosci. 13:84. doi: 10.3389/fnins.2019.00084, 30872985 PMC6403189

[ref12] Eldaief M. C. Halko M. A. Buckner R. L. Pascual-Leone A. (2011). Transcranial magnetic stimulation modulates the brain's intrinsic activity in a frequency-dependent manner. Proc. Natl. Acad. Sci. 108, 21229–21234. doi: 10.1073/pnas.1113103109, 22160708 PMC3248528

[ref13] Fox M. D. Buckner R. L. White M. P. Greicius M. D. Pascual-Leone A. (2012). Efficacy of transcranial magnetic stimulation targets for depression is related to intrinsic functional connectivity with the subgenual cingulate. Biol. Psychiatry 72, 595–603. doi: 10.1016/j.biopsych.2012.04.028, 22658708 PMC4120275

[ref14] Fox M. D. Halko M. A. Eldaief M. C. Pascual-Leone A. (2012). Measuring and manipulating brain connectivity with resting state functional connectivity magnetic resonance imaging (fcMRI) and transcranial magnetic stimulation (TMS). NeuroImage 62, 2232–2243. doi: 10.1016/j.neuroimage.2012.03.035, 22465297 PMC3518426

[ref15] Franke L. M. Gitchel G. T. Perera R. A. Hadimani R. L. Holloway K. L. Walker W. C. (2022). Randomized trial of rTMS in traumatic brain injury: improved subjective neurobehavioral symptoms and increases in EEG delta activity. Brain Inj. 36, 683–692. doi: 10.1080/02699052.2022.2033845, 35143365

[ref16] He D. Kang Q. Duan W. Li G. He R. Liu X. . (2025). Investigating the role of gut-derived neurotoxin TMAO in PTSD risk following traumatic brain injury. Actas Esp. Psiquiatr. 53, 730–741. doi: 10.62641/aep.v53i4.1885, 40791048 PMC12353233

[ref17] Kim H. Choi J. Jeong B. Fava M. Mischoulon D. Park M. J. . (2022). Impaired oxygenation of the prefrontal cortex during verbal fluency task in young adults with major depressive disorder and suicidality: a functional near-infrared spectroscopy study. Front. Psych. 13:915425. doi: 10.3389/fpsyt.2022.915425, 35815016 PMC9260011

[ref18] Lindsey A. Ellison R. L. Herrold A. A. Aaronson A. L. Kletzel S. L. Stika M. M. . (2023). rTMS/iTBS and cognitive rehabilitation for deficits associated with TBI and PTSD: a theoretical framework and review. J. Neuropsychiatry Clin. Neurosci. 35, 28–38. doi: 10.1176/appi.neuropsych.21090227, 35872613

[ref19] Mihara M. Miyai I. (2016). Review of functional near-infrared spectroscopy in neurorehabilitation. Neurophotonics. 3:031414. doi: 10.1117/1.NPh.3.3.031414, 27429995 PMC4940623

[ref20] Neville I. S. Zaninotto A. L. Hayashi C. Y. Rodrigues P. A. Galhardoni R. Ciampi de Andrade D. . (2019). Repetitive TMS does not improve cognition in patients with TBI: a randomized double-blind trial. Neurology 93, e190–e199. doi: 10.1212/WNL.0000000000007748, 31175209 PMC6656650

[ref21] Nousia A. Martzoukou M. Liampas I. Siokas V. Bakirtzis C. Nasios G. . (2022). The effectiveness of non-invasive brain stimulation alone or combined with cognitive training on the cognitive performance of patients with traumatic brain injury: α systematic review. Arch. Clin. Neuropsychol. 37, 497–512. doi: 10.1093/arclin/acab047, 34155517

[ref22] Obasa A. A. Olopade F. E. Juliano S. L. Olopade J. O. (2024). Traumatic brain injury or traumatic brain disease: a scientific commentary. Brain Multiphys. 6:100092. doi: 10.1016/j.brain.2024.100092

[ref23] Polanía R. Nitsche M. A. Ruff C. C. (2018). Studying and modifying brain function with non-invasive brain stimulation. Nat. Neurosci. 21, 174–187. doi: 10.1038/s41593-017-0054-4, 29311747

[ref24] Premoli I. Castellanos N. Rivolta D. Belardinelli P. Bajo R. Zipser C. . (2014). TMS-EEG signatures of GABAergic neurotransmission in the human cortex. J. Neurosci. 34, 5603–5612. doi: 10.1523/JNEUROSCI.5089-13.2014, 24741050 PMC6608220

[ref25] Prokop-Millar S. Di Passa A.-M. Yaya H. McIntyre-Wood C. Farzan F. Terpstra A. . (2025). Predictive and mechanistic biomarkers of treatment response to transcranial magnetic stimulation (TMS) in psychiatric and neurocognitive disorders, identified via TMS-electroencephalography (EEG) and resting-state EEG: a systematic review. J. Affect. Disord. 393:120194. doi: 10.1016/j.jad.2025.120194 40925485

[ref26] Rossi S. Hallett M. Rossini P. M. Pascual-Leone A. Group SoTC (2009). Safety, ethical considerations, and application guidelines for the use of transcranial magnetic stimulation in clinical practice and research. Clin. Neurophysiol 120, 2008–2039. doi: 10.1016/j.clinph.2009.08.016, 19833552 PMC3260536

[ref27] Trandafir D. M. Zamfirache F. Dumitru C. Radu B. M. Ciobanu A. M. (2026). Repetitive transcranial magnetic stimulation in major depressive disorder: from bench to bedside—a scoping review of neurobiological mechanisms and clinical translation. Bioengineering 13:288. doi: 10.3390/bioengineering13030288, 41899819 PMC13023697

[ref28] Vitello M. M. Rosenfelder M. J. Cardone P. Niimi M. Willacker L. Thibaut A. . (2023). A protocol for a multicenter randomized and personalized controlled trial using rTMS in patients with disorders of consciousness. Front. Neurol. 14:1216468. doi: 10.3389/fneur.2023.1216468, 37545735 PMC10401598

[ref29] Zhan X. Zhou Z. Liu Y. Cecchi N. J. Hajiahamemar M. Zeineh M. M. . (2024). Differences between two maximal principal strain rate calculation schemes in traumatic brain analysis with in-vivo and in-silico datasets. arXiv 1:e08164. doi: 10.48550/arXiv.2409.08164 39671828

[ref30] Zhang J. J. Bai Z. Fong K. N. (2024). Methodological considerations of priming repetitive transcranial magnetic stimulation protocols in clinical populations. Gen. Psychiatry 37:e101237. doi: 10.1136/gpsych-2023-101237, 38317830 PMC10840021

